# PD-L1 siRNA Theranostics With a Dextran Nanoparticle Highlights the Importance of Nanoparticle Delivery for Effective Tumor PD-L1 Downregulation

**DOI:** 10.3389/fonc.2020.614365

**Published:** 2021-02-25

**Authors:** Jesus Pacheco-Torres, Marie-France Penet, Balaji Krishnamachary, Yelena Mironchik, Zhihang Chen, Zaver M. Bhujwalla

**Affiliations:** ^1^ Division of Cancer Imaging Research, The Russell H. Morgan Department of Radiology and Radiological Science, The Johns Hopkins University School of Medicine, Baltimore, MD, United States; ^2^ Sidney Kimmel Comprehensive Cancer Center, The Johns Hopkins University School of Medicine, Baltimore, MD, United States; ^3^ Department of Radiation Oncology and Molecular Radiation Sciences, The Johns Hopkins University School of Medicine, Baltimore, MD, United States

**Keywords:** siRNA, PD-L1, theranostics, imaging, biodegradable dextran, PD-L1 downregulation

## Abstract

**Purpose:**

The inhibition of immune checkpoints such as programmed cell death ligand-1 (PD-L1/CD274) with antibodies is providing novel opportunities to expose cancer cells to the immune system. Antibody based checkpoint blockade can, however, result in serious autoimmune complications because normal tissues also express immune checkpoints. As sequence-specific gene-silencing agents, the availability of siRNA has significantly expanded the specificity and range of “druggable” targets making them promising agents for precision medicine in cancer. Here, we have demonstrated the ability of a novel biodegradable dextran based theranostic nanoparticle (NP) to deliver siRNA downregulating PD-L1 in tumors. Optical imaging highlighted the importance of NP delivery and accumulation in tumors to achieve effective downregulation with siRNA NPs, and demonstrated low delivery and accumulation in several PD-L1 expressing normal tissues.

**Methods:**

The dextran scaffold was functionalized with small molecules containing amine groups through acetal bonds. The NP was decorated with a Cy5.5 NIR probe allowing visualization of NP delivery, accumulation, and biodistribution. MDA-MB-231 triple negative human breast cancer cells were inoculated orthotopically or subcutaneously to achieve differences in vascular delivery in the tumors. Molecular characterization of PD-L1 mRNA and protein expression in cancer cells and tumors was performed with qRT-PCR and immunoblot analysis.

**Results:**

The PD-L1 siRNA dextran NPs effectively downregulated PD-L1 in MDA-MB-231 cells. We identified a significant correlation between NP delivery and accumulation, and the extent of PD-L1 downregulation, with *in vivo* imaging. The size of the NP of ~ 20 nm allowed delivery through leaky tumor vasculature but not through the vasculature of high PD-L1 expressing normal tissue such as the spleen and lungs.

**Conclusions:**

Here we have demonstrated, for the first time, the feasibility of downregulating PD-L1 in tumors using siRNA delivered with a biodegradable dextran polymer that was decorated with an imaging reporter. Our data demonstrate the importance of tumor NP delivery and accumulation in achieving effective downregulation, highlighting the importance of imaging in siRNA NP delivery. Effective delivery of these siRNA carrying NPs in the tumor but not in normal tissues may mitigate some of the side-effects of immune checkpoint inhibitors by sparing PD-L1 inhibition in these tissues.

## Introduction

The identification of immune checkpoints such as PD-L1 is providing exciting new advances in cancer treatments designed to block these checkpoints, exposing cancer cells to the immune system. Antibody based checkpoint blockade can, however, result in serious autoimmune complications such as vitiligo, colitis, and lupus (1). Furthermore, recent reports have identified additional roles of PD-L1 in cancer cells within intracellular compartments that are not accessible by antibodies (2). As sequence-specific gene-silencing agents, siRNA have significantly expanded the specificity and range of “druggable” targets making them promising agents for precision medicine in cancer (3). siRNAs are being actively investigated as molecular-based therapeutic strategies in clinical trials (4) in several diseases including lipid disorders (5), neurological diseases (6, 7), cancer (8), and cardiovascular diseases (9). Several NPs have been developed for effective siRNA delivery (3). By decorating these NPs with an imaging reporter, it is possible to visualize the delivery and distribution of the NPs in the tumor for theranostics. Imaging these NPs allows an evaluation of the role of NP delivery in downregulation of the target gene. NPs of ~20 nm in diameter extravasate into tumors through leaky tumor vasculature, but do not easily extravasate through normal vasculature (10–12). This is important for most tumors where specific receptors or antigens are not available for targeting (13). Effective delivery of these siRNA carrying NPs within the tumor, but not in normal tissues, would mitigate some of the side-effects of immune checkpoint inhibitors.

We previously synthesized an imaging reporter labeled biodegradable dextran NP to use as an efficient cationic polymer carrier for siRNA delivery (14). As a homopolysaccharide of glucose, dextran has been used as a drug carrier in human applications due to its biodegradability, wide availability, and ease of modification (15). For electrostatic binding with siRNA, necessary amine functional groups are conjugated to the dextran platform through acetal bonds. Acetals are attractive for the release of therapeutic cargo through cleavage of the bond under acidic conditions that occur in cancer and inflammation, as well as within endocytosis compartments (16, 17). When the NP was delivered within cancer cells, the acetal bond was cleaved under weak acid conditions that was clearly visualized through the use of multiple imaging reporters (14). The rapid cleavage and release of amine groups minimized the proinflammatory side effects of the positively charged amine groups making this cationic nanopolymer a useful carrier for siRNA delivery to downregulate gene expression. The siRNA is bound electrostatically to the amine groups. Transmission electronic microscopy (TEM) imaging identified a diameter of approximately 20 nm.

Here we report, for the first time, on the use of this theranostic dextran NP to deliver PD-L1 siRNA in triple negative MDA-MB-231 human breast cancer xenografts. Tumors were inoculated orthotopically or subcutaneously as orthotopic tumors are better vascularized than subcutaneous tumors (18, 19), allowing us to evaluate the role of NP delivery in downregulation of PD-L1. Optical imaging was used to visualize the delivery and biodistribution of the NP *in vivo*. Molecular characterization established the downregulation of PD-L1 message and protein in the tumors. Image analysis of the NP delivery showed a close association between NP delivery and downregulation of PD-L1, highlighting the importance of NP delivery in target downregulation, and the importance of noninvasive imaging in determining NP delivery to establish effectiveness of target gene downregulation.

## Materials and Methods

### Synthesis of PD-L1 siRNA Dextran Nanoparticles

The PD-L1 siRNA dextran NP was synthesized as previously described (20) with a few modifications. Briefly, the dextran (70 kDa) scaffold was reacted with an excess of N-(2-(bis(2-aminoethyl)amino)ethyl)-4-(4-(dimethoxymethyl)-2-methoxyphenoxy)butanamide to attach the amine groups to the dextran polymer through the acetal bonds. ^1^H NMR spectra indicated that the functionalized degree of glucose residues was around 0.35. Then Cy5.5 was conjugated to amine groups on the dextran platform (approximately 1 Cy5.5 molecule per dextran molecule).

This modified dextran scaffold was mixed with siRNA in reduced serum medium (ThermoFisher, Waltham, MA, USA) for cell studies, or with PBS for *in vivo* studies, for 20 min immediately prior to adding to cell culture or prior to injecting into mice. All the siRNA dextran NPs contained a ratio of nitrogen atoms in one dextran molecule to phosphor atoms in one siRNA molecule (N/P ratio) equal to 15.

### Cell Culture

Human breast cancer MDA-MB-231 cells were obtained from American Type Culture Collection (ATCC) (Manassas, VA, USA). Fetal bovine serum, penicillin, and streptomycin were from Invitrogen (Carlsbad, CA, USA). Cells were maintained in RPMI 1640 (Invitrogen, Grand Island, NY, USA) supplemented with 10% fetal bovine serum in a humidified incubator at 37°C/5% CO_2_. Cells were seeded at a density of 400,000 cells per dish in 60 mm dish (for qRT-PCR experiments) or 1,000,000 cells per dish in 100 mm dish (for immunoblots experiments) 24 h prior to the transfection experiment.

### Cell Studies With PD-L1 siRNA Dextran Nanoparticles

All siRNAs were purchased from Dharmacon (Lafayette, CO, USA). Untreated cells and cells treated with non-targeting scrambled siRNA (Dharmacon, Catalog Item D-001810-10-20) were used as controls. Isoform‐specific siRNA was custom designed using Thermo Scientific siRNA Design Center (Thermo Scientific, Rockford, IL, USA). siRNA specific sequence was 5’-GAGGAAGACCUGAAGGUUCAGCAUA-3’ for PD-L1. For scrambled siRNA, we used the commercial ON-TARGETplus Non-targeting Control Pool (catalog number D-001810-10-20) comprised of the following siRNA sequences: 5’-UGGUUUACAUGUCGACUAA-3’, 5’-UGGUUUACAUGUUGUGUGA-3’, 5’-UGGUUUACAUGUUUUCUGA-3’ and 5’-UGGUUUACAUGUUUUCCUA-3’.

Cells were incubated for 48 h in RPMI 1640 medium containing siRNA-PD-L1 dextran NPs (concentration of siRNA: 100 pmol/mL, N/P = 15). Cells were treated with NPs for 48 h, because this incubation period resulted in the most effective downregulation of the target genes. All transfections were carried out based on established protocols (20).

### Mouse Model and Tumor Implantation

All *in vivo* studies were done in compliance with guidelines established by the Institutional Animal Care and Use Committee of the Johns Hopkins University. MDA-MB-231 human breast cancer cells (2 × 10^6^ cells/mouse) were inoculated orthotopically in the mammary fat pad (n = 15) or subcutaneously (n = 10) in female severe combined immunodeficient (SCID) mice. Tumors were palpable within two to three weeks after implantation and reached a volume of approximately 300–400 mm^3^ within four to five weeks, at which time they were used for the studies.

### *In Vivo* RNA Interference Experiments

For biodistribution studies, MDA-MB-231 tumor bearing mice were injected intravenously with 200 µl of PD-L1 siRNA dextran NPs (PD-L1siRNA, 5 nmol/mouse/dose; dextran 2.5 mg/mouse/dose, N/P = 15) through the tail vein. Two different protocols were tested. In group 1, mice received two doses of NPs 48 h apart and were sacrificed at 24 h after the second injection (n = 10, four orthotopic, six subcutaneous). In group 2, mice received two doses 48 h apart, but were sacrificed at 48 h after the second injection (n = 5, orthotopic). A group of 10 mice were injected with an equivalent volume of PBS and served as controls.

### *In Vivo* and *Ex Vivo* Optical Imaging Studies

*In vivo* and *ex vivo* optical images were acquired with a Pearl^®^ Trilogy Small Animal Imaging System (LI-COR, Lincoln, NE). Delivery of the NPs was confirmed by imaging the mice *in vivo* at 48 h after the first injection and either at 24 h (group 1) or at 48 h (group 2) after the second injection. Mice were sacrificed and organs excised for *ex vivo* quantification. Excised tumors, kidneys, liver, spleen, heart, lungs, and muscle were imaged. Fluorescent intensities in regions of interest (ROIs) were quantified by using Living Image 4.5 software (Caliper, Hopkinton, MA). The tumors were sectioned into two to three slices of ~1 mm thickness. Fluorescent signal was acquired from both sides of each slice, and the values acquired for each tumor were averaged. Signal intensities were normalized to the area of the ROI.

### RNA Isolation and Quantitative Reverse Transcription-PCR

Total RNA was isolated from MDA-MB-231 cells grown in 60 mm dish or from frozen MDA-MB-231 tumor tissue by using QIAshredder and RNeasy Mini kit (Qiagen, Valencia, CA, USA) as per the manufacturer’s protocol. cDNA was prepared using the iScript cDNA synthesis kit (Bio-Rad, Hercules, CA, USA). cDNA samples were diluted at 1:10 dilution and quantitative real-time PCR was performed using IQ SYBR Green supermix and gene specific primers in the iCycler real-time PCR detection system (Bio-Rad). All primers were designed using either Beacon designer software 7.8 (premier Biosoft, Palo Alto, CA, USA) or publicly available Primer3plus software. The expression of target RNA relative to the housekeeping gene hypoxanthine phosphoribosyltransferase 1 (HPRT1) was calculated based on the threshold cycle (C_t_) as R = 2-^Δ(ΔCt)^, where ΔC_t_ = C_t_ of target gene - C_t_ of HPRT1 and Δ(ΔC_t_)= ΔC_t_ siRNA treated cells/tumor - ΔC_t_ untreated cells/tumors.

### Protein Isolation and Immunoblots

Total protein was extracted from MDA-MB-231 cells grown in a 100 mm dish or from frozen MDA-MB-231 tumor tissue by using 1x cracking buffer [100 mmol/L Tris (pH 6.7), 2% glycerol] containing a protease inhibitor (Sigma) at 1:200 dilution. Protein concentration was estimated using the Bradford Bio-Rad protein assay Kit (Bio-Rad). Approximately 100 µg of total protein was used in each experiment. Expression levels of PD-L1 were determined by immunoblotting using a rabbit polyclonal against human PD-L1 at 1:1,000 dilution (GeneTex, Irvine, CA). Monoclonal anti-GAPDH antibody (1:50,000 dilution, Sigma-Aldrich) was used as loading control. Proteins were visualized with HRP (horseradish peroxidase)-conjugated secondary antibodies using the SuperSignal West Pico Chemiluminescent substrate kit (Thermo Scientific).

### Statistical Analysis

Statistical analyses were performed using GraphPad Prism 4 software (GraphPad Software, Inc., San Diego, CA, USA). To determine the statistical significance of the quantified data, an unpaired two-tailed Student’s T-test was performed. P values ≤0.05 were considered significant unless otherwise stated.

## Results

### Effective PD-L1 Downregulation in Cells Following Treatment With PD-L1 siRNA Dextran Nanoparticles

**Figure 1:**
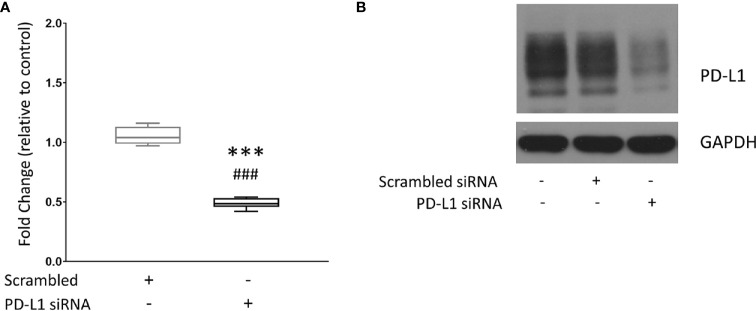
Downregulation of PD-L1 in MDA-MB-231 cells. **(A)** Relative fold change of PD-L1 messenger RNA (mRNA) expression in MDA-MB-231 cells. Fold changes were normalized to untreated cells. Values are represented in box and whisker format with minimum to maximum values from 4–7 independent experiments. **(B)** Representative immunoblot assays of PD-L1 (top) and GAPDH (bottom) following PD-L1 siRNA dextran NP incubation. MDA-MB-231 cells were untreated (control) or transfected with either 100 nM scrambled siRNA/dextran, or 100 nM PD-L1 siRNA/dextran (N/P = 15). ***p ≤ 0.001 compared to untreated cells. ^###^p ≤ 0.001 compared to cells transfected with scrambled siRNA.

MDA-MB-231 triple negative human breast cancer cells were treated with the dextran NP as a carrier for PD-L1 siRNA. Quantitative reverse transcription polymerase chain reaction (qRT-PCR) was performed to measure PD-L1 expression in untreated MDA-MB-231 cells, and in MDA-MB-231 cells treated with scrambled siRNA dextran NPs used as controls or treated with PD-L1 siRNA dextran NPs. Values were normalized to mRNA levels measured in untreated cells. **Figure 1A** shows changes in mRNA levels in PD-L1 siRNA dextran NP treated cells, compared to scrambled siRNA NP treated cells. Treatment with scrambled siRNA NPs did not alter PD-L1 mRNA levels. A significant decrease of ~50% PD-L1 mRNA was detected in cells following treatment with PD-L1 siRNA dextran NPs compared to treatment with scrambled siRNA dextran NPs. To determine whether changes in mRNA translated to changes in PD-L1 protein levels, we analyzed proteins obtained from untreated cells, and cells treated with scrambled siRNA dextran NPs or with PD-L1 siRNA dextran NPs, by immunoblotting. As shown in **Figure 1B**, PD-L1 siRNA dextran NP treatment resulted in an effective decrease of the PD-L1 protein. There were no changes in PD-L1 protein in cells treated with scrambled siRNA dextran NPs compared to untreated cells.

### *In Vivo* and *Ex Vivo* Imaging of PD-L1 siRNA Dextran Nanoparticles in MDA-MB-231 Tumors and Organs

**Figure 2:**
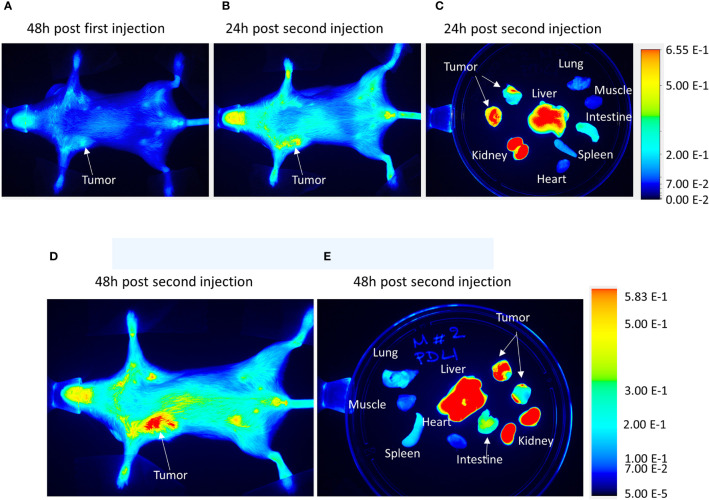
*In vivo* PD-L1 siRNA dextran NP biodistribution. Representative *in vivo* images acquired from an orthotopic MDA-MB-231 tumor bearing mouse **(A)** 48 h after the first injection of PD-L1 siRNA dextran NPs, **(B)** 24 h after a second injection of PD-L1-siRNA-dextran NPs, and **(C)** the corresponding *ex vivo* images. Representative *in vivo* images acquired from an orthotopic MDA-MB-231 tumor bearing mouse **(D)** 48 h after a second injection of PD-L1-siRNA-dextran NPs and **(E)** the corresponding *ex vivo* images.

**Figure 3:**
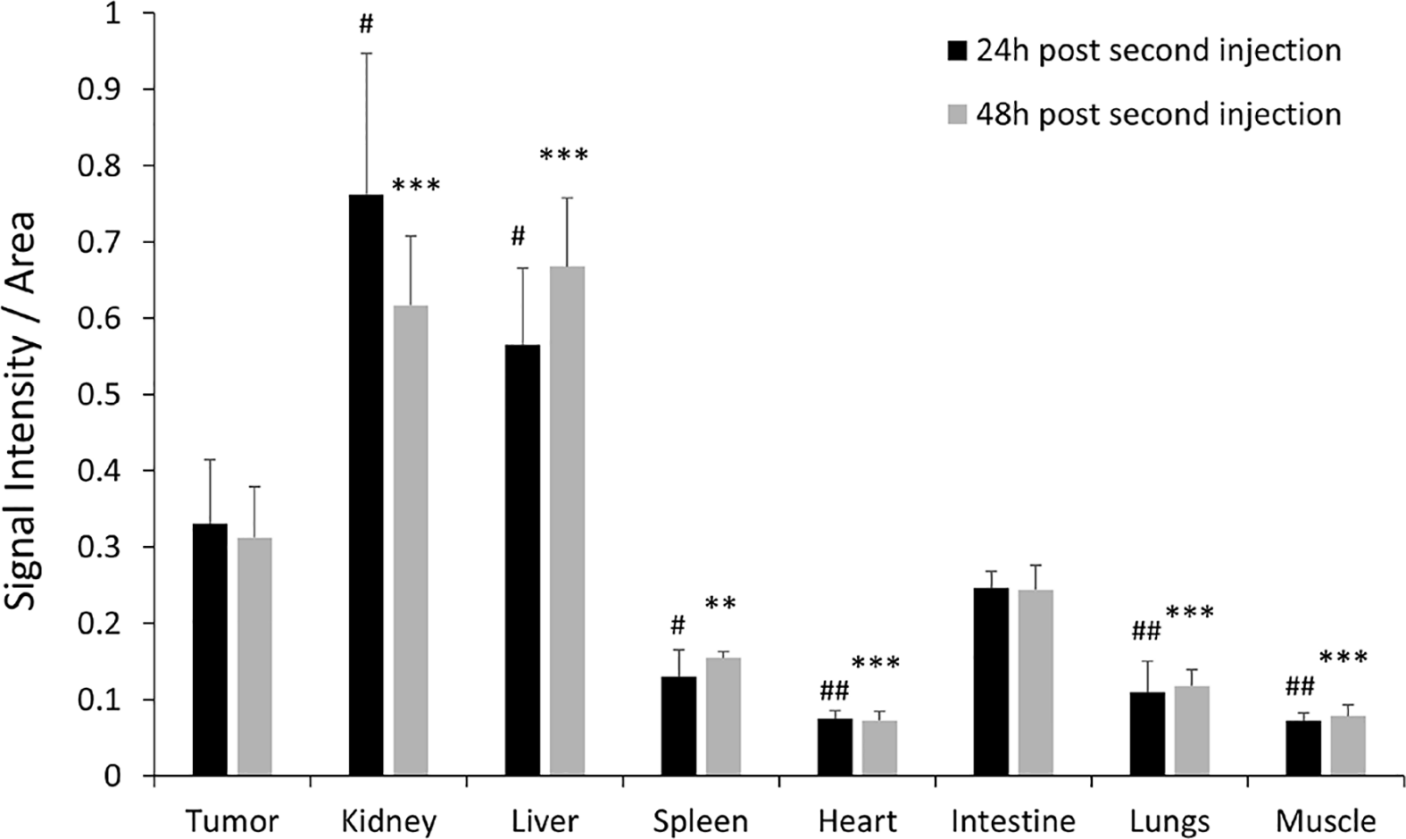
*Ex vivo* PD-L1 siRNA dextran NP biodistribution in orthotopic tumors. Quantification of fluorescent signal intensity in tumors, kidneys, liver, spleen, lungs, heart, intestine, and muscle measured *ex vivo* after PD-L1-siRNA dextran NP treatment 24 h after the second injection (group 1, black bars, n = 4) and 48 h after the second injection (group 2, grey bars, n = 5). ^#^p < 0.05, ^##^p < 0.01 (compared to the signal measured in the tumor 24 h post second injection). **p < 0.01, ***p < 0.005 (compared to the signal measured in the tumor 48 h post second injection).

**Figure 4:**
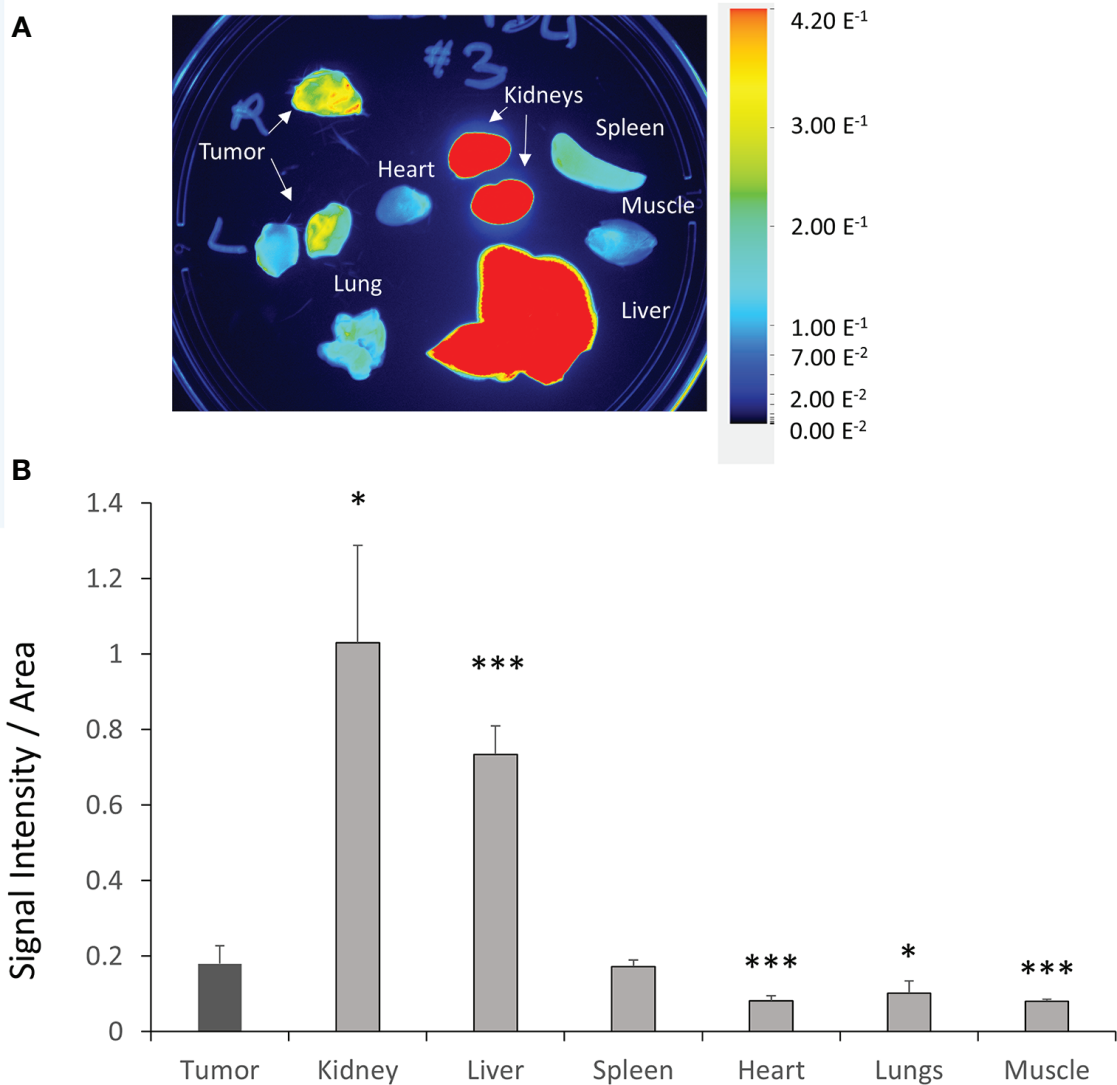
*Ex vivo* PD-L1 siRNA dextran NP biodistribution in subcutaneous tumors. **(A)** Representative *ex vivo* images acquired from subcutaneous MDA-MB-231 tumors, kidneys, liver, spleen, lungs, heart, intestine, and muscle 24 h after the second injection of PD-L1-siRNA-dextran NPs. **(B)** Quantification of fluorescent signal in subcutaneous MDA-MB-231 tumors, kidneys, liver, spleen, lungs, heart, intestine, and muscle measured *ex vivo* 24 h after the second PD-L1-siRNA dextran NP injection (group 1, n = 6). *p < 0.05, ***p < 0.005 compared to the fluorescent signal measured in the tumor.

To validate the efficacy of siRNA delivery of the PD-L1 siRNA dextran NPs and the role of NP delivery in PD-L1 downregulation, we established orthotopic mammary fat pad and subcutaneous MDA-MB-231 xenografts. Cy5.5 labeled PD-L1 siRNA dextran NPs were injected through the tail vein and *in vivo* and *ex vivo* distribution of the NPs was confirmed using the fluorescent signal from the NIR optical reporter Cy5.5 attached to the dextran (20). Mice received two doses of the NP given 48 h apart and were sacrificed at 24 h (group 1) or 48 h (group 2) after the second dose. NP accumulation in orthotopic MDA-MB-231 tumors was observed *in vivo* at 48 h after injection of the first dose as shown in the representative image in **Figure 2A**. Representative *in vivo* images obtained at 24 h or at 48 h following *i.v.* administration of the second dose are presented in **Figure 2B** and **Figure 2D**, respectively. As observed in these representative images, NPs were also detected in the liver *in vivo*. Corresponding representative *ex vivo* images obtained at 24 h after the second dose and at 48 h after the second dose are presented in **Figure 2C** and **Figure 2E**, respectively. The *ex vivo* images confirmed the increased NP uptake in the tumors with a tumor to muscle ratio (T/M) of approximately four. The optical images demonstrated the heterogeneity of the NP distribution within the tumor both *in vivo* and in tumor sections *ex vivo*, due to the heterogeneity of tumor vasculature. Biodistribution data of the NPs in orthotopic tumors and different organs obtained at 24 and 48 h after the second dose are summarized for nine mice in **Figure 3**. NP accumulation in these tumors was comparable at 24 and 48 h. Representative *ex vivo* images obtained from subcutaneous tumors that are less vascularized than orthotopic tumors are presented in **Figure 4A**. Biodistribution data of the NPs in subcutaneous tumors and different organs obtained at 24 h after the second dose are summarized for six tumors in **Figure 4B**. A significantly lower tumor retention and a lower tumor to muscle ratio, of approximately 2.3, was observed in subcutaneous tumors compared to orthotopic tumors.

Compared to orthotopic tumors, NP accumulation in spleen, heart, lungs, and muscle was significantly lower (**Figure 3**). As anticipated, since NPs of this size are cleared by the reticuloendothelial system, we found significantly higher accumulation in the liver.

### Downregulation of PD-L1 in Tumors

**Figure 5:**
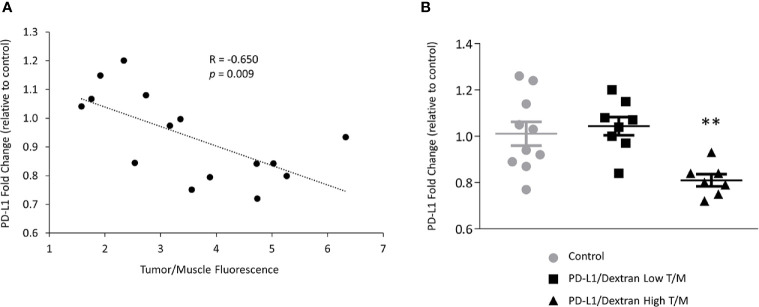
*In vivo* downregulation of PD-L1 in MDA-MB-231 tumors treated with PD-L1 siRNA dextran. **(A)** Correlation between the levels of PD-L1 mRNA, represented by PD-L1 fold change relative to control tumors, and the tumor/muscle fluorescence ratio in tumors treated with PD-L1 siRNA dextran NPs. **(B)** Relative fold change of PD-L1 mRNA expression in MDA-MB-231 tumors treated with the PD-L1 siRNA dextran NPs. Mice were injected with PBS, or PD-L1 siRNA dextran NPs through the tail vein. Animals were divided, according to the tumor to muscle fluorescence ratio measured *in vivo*, into the highest (triangles) and lowest (squares) 50% values. Fold changes were normalized to tumors treated with PBS. Lines represent Mean ± Standard Error of the Mean. **p < 0.01 compared to control tumors or low tumor to muscle fluorescence ratio tumors.

**Figure 6:**
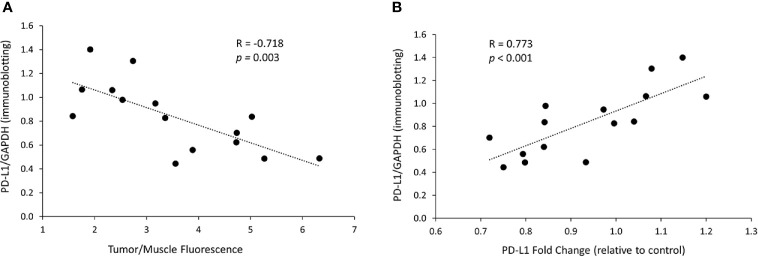
*In vivo* PD-L1 protein levels decrease is related to dextran accumulation in the tumor and to mRNA changes. **(A)** Correlation between the PD-L1 protein levels, represented by immunoblotting intensity ratio of PD-L1 to GAPDH, and the tumor/muscle fluorescence ratio in tumors treated with the siRNA/dextran complex. **(B)** Correlation between the protein levels of PD-L1, represented by immunoblotting intensity ratio of PD-L1 to GAPDH, and the levels of PD-L1 mRNA, represented by PD-L1 fold change relative to control, in tumors treated with the siRNA/dextran complex.

The importance of siRNA NP delivery and accumulation in the effectiveness of PD-L1 downregulation is highlighted in **Figure 5** for mRNA and **Figure 6** for protein expression. A significant correlation (R = -0.650, p = 0.009) between the fold-decrease of PD-L1 mRNA normalized to control values, and the tumor/muscle fluorescence was observed as shown in **Figure 5A** demonstrating that tumors with higher NP delivery showed a greater reduction of PD-L1. This dependence was further confirmed when we separated the tumors into the highest 50% and lowest 50% tumor/muscle fluorescence groups. The highest 50% group consisted of seven orthotopic tumors, three from group 1 and four from group 2. The lowest 50% group consisted of six subcutaneous and two orthotopic tumor, all from group 1. We found a significant reduction in fold change of PD-L1 mRNA in the tumors with high tumor/muscle fluorescence compared to control tumors as shown in **Figure 5B**. In tumors with low tumor/muscle fluorescence, there was no significant difference in PD-L1 mRNA compared to control tumors.

We next analyzed the relationship between PD-L1 protein expression and the NP delivery and accumulation in the tumor. PD-L1 proteins levels were represented by the PD-L1/GAPDH ratio measured in immunoblots. The accumulation of PD-L1 siRNA dextran NPs in the tumor was represented by the tumor/muscle fluorescence ratio. As shown in **Figure 6A**, we observed that the decrease in PD-L1 protein levels in tumors directly correlated with the tumor/muscle fluorescence ratio (R = -0.718, *p* = 0.003). Additionally, we observed a significant correlation (R = 0.773, *p* < 0.001) between the PD-L1 protein levels and the mRNA levels as shown in **Figure 6B**, confirming that the effective decrease of mRNA translated to an effective decrease of protein in these tumors.

## Discussion

Our purpose in these studies was to demonstrate the ability of the dextran siRNA NPs to downregulate PD-L1 in tumors, and to highlight the importance of siRNA NP delivery and accumulation in achieving effective downregulation. We established that PD-L1 siRNA dextran NPs could downregulate PD-L1 in tumors, provided that effective NP delivery and accumulation were achieved. Based on the biodistribution studies we found that NP accumulation was significantly lower in spleen, heart, lungs, and muscle compared to the tumors. Because NPs of this size are cleared by the reticuloendothelial system (RES), we found significantly higher accumulation in the liver. Renal accumulation of the NPs was likely due to renal clearance of molecules (21).

Antibody-based immunotherapies target normal tissues where the immune checkpoint is expressed along with the tumor. This leads to significant side-effects (22, 23). As a result, novel approaches to targeting immune checkpoints using small molecules, peptides and macrocycles, are being actively explored (24, 25). According to the Human Protein Atlas (26), in addition to cancer cells, PD-L1 is also expressed in healthy lungs, heart, colon, and spleen. Inhibiting PD-L1 in these organs can lead to immune-related pneumonitis, myocarditis, and colitis (27, 28). The use of siRNA NPs that primarily accumulate in tumors but not in normal tissues would reduce side-effects associated with immune checkpoint inhibition in normal tissues. In addition, the use of siRNA NPs provides the potential to combine multiple siRNAs directed toward different molecular pathways, including multiple immune checkpoints, within a single NP (3). This is especially significant as studies have shown the impact of tumor metabolism on PD-L1 levels (29–31) and the immune response (32–34). As a result, metabolic inhibitors of different pathways are being evaluated in clinical trials in combination with immune-checkpoint inhibitors, with promising outcomes (35). This creates the possibility of including siRNA that downregulate enzymes in metabolic pathways in combination with immune checkpoint siRNA. In addition, PD-L1 has pro-oncogenic roles beyond its traditional functions in immunomodulation making its downregulation important (36–41).

Several nanopolymers have been evaluated as molecular agents to deliver PD-L1 siRNA in tumor. A polymeric carrier consisting of disulfide-cross-linked polyethylenimine and dermatan sulfate was used to deliver PD-L1 siRNA *in vivo* in a mouse melanoma model (42). In another study, PLGA NPs simultaneously delivered PD-1 and PD-L1 siRNA, silencing these genes in cytotoxic T lymphocytes and tumor cells in a colon murine model (43). A polymer containing a poly-L-lysine-lipoic acid reduction-sensitive core and a tumor extracellular pH-stimulated shedding polyethylene glycol layer was used to co-deliver PD-L1 siRNA and doxorubicin in a melanoma model with promising results (44). Silencing the expression of PD-L1 in dendritic cells (DCs) and of PD-1 in T cells by siRNA-loaded chitosan-dextran sulfate nanoparticles was recently described (45). *Ex vivo* evaluation of the DC phenotypic and functional characteristics, and of the T-cell functions following tumor antigen recognition on DCs, showed that PD-L1-silenced DCs presented a potent immunotherapeutic approach in combination with PD-1 siRNA loaded NPs.

Here, we reported, for the first time, the use of a biodegradable dextran nanopolymer as an siRNA carrier to selectively downregulate PD-L1 in a xenograft model of triple negative human breast cancer. With imaging, we demonstrated that the siRNA NPs successfully accumulated in tumors to downregulate PD-L1 expression. Cancer cells induce neovascularization by co-opting and remodeling existing vasculature (46), by stimulating angiogenesis and inducing sprouting of new blood capillaries from existing ones (47, 48). This vasculature is chaotic and heterogeneous (49), contributing significantly to heterogeneities in NP delivery and accumulation. Our imaging data highlight the importance of being able to detect NP delivery within tumors, and the importance of NP delivery and accumulation in NP function. Integrating imaging into siRNA NP delivery will allow optimization of NP structure and tumor manipulation to improve delivery. One of the limitations of this study is the use of fluorescence imaging to detect NP accumulation in the tumor, as this is not translatable to human applications. This limitation can be overcome in future studies by decorating the NP with a radiolabel, or with an MR contrast agent, so that the biodistribution and delivery can be detected in deep-seated tumors and tissues with nuclear imaging or MRI.

## Conclusion

Our data demonstrate that, while it is possible to significantly downregulate tumor PD-L1 with siRNA NPs, effective delivery is critically important in achieving effective downregulation. Imaging can play an important role in tracking effective delivery of siRNA NPs *in vivo*. Strategies to improve siRNA NP delivery should continue to be an area of major emphasis. The siRNA NP strategy targeting PD-L1 presented here has several advantages over traditional antibodies or pharmacological based therapies. It can be made tumor specific to reduce side effects and can be multiplexed with multiple siRNA targeting other pro-oncogenic pathways. Downregulation with siRNA can also reduce the pro-oncogenic roles of immune checkpoints, by limiting *de novo* synthesis. The dextran-based PD-L1 siRNA NP showed significant downregulation of PD-L1 expression in tumors where effective delivery was achieved. Because of its biocompatibility and synthesis reproducibility, this siRNA carrier has a clear path for translational applications to achieve effective PD-L1 or other siRNA delivery in patients.

## Data Availability Statement

The raw data supporting the conclusions of this article will be made available by the authors, without undue reservation.

## Ethics Statement

The animal study was reviewed and approved by The Institutional Animal Care and Use Committee of the Johns Hopkins University.

## Author Contributions

All authors conceptualized and designed the study. ZC performed the Dextran NP synthesis, purification, and characterization. BK, M-FP, YM, and JP-T collected and assembled the data. BK, M-FP, JP-T, YM, ZC, and ZB analyzed and interpreted the data. All authors contributed to the article and approved the submitted version.

## Funding

The study was supported by NIH Grant/Award Numbers: R01 CA253617, P30 CA006973, P41 EB024495, R01 CA82337, R35 CA209960. JP-T was funded by Fundación Alonso Martín Escudero and Marie Skłodowska-Curie Actions.

## Conflict of Interest

The authors declare that the research was conducted in the absence of any commercial or financial relationships that could be construed as a potential conflict of interest.

## References

[1] Spain L Diem S Larkin J. Management of toxicities of immune checkpoint inhibitors. Cancer Treat Rev (2016) 44:51–60. 10.1016/j.ctrv.2016.02.001 26874776

[2] Wu Y Chen W Xu ZP Gu W. PD-L1 Distribution and Perspective for Cancer Immunotherapy-Blockade, Knockdown, or Inhibition. Front Immunol (2019) 10:2022. 10.3389/fimmu.2019.02022 31507611 PMC6718566

[3] Chen Z Krishnamachary B Pachecho-Torres J Penet MF Bhujwalla ZM. Theranostic small interfering RNA nanoparticles in cancer precision nanomedicine. Wiley Interdiscip Rev Nanomed Nanobiotechnol (2020) 12(2):e1595. 10.1002/wnan.1595 31642207 PMC7360334

[4] Crooke ST Witztum JL Bennett CF Baker BF. RNA-Targeted Therapeutics. Cell Metab (2018) 27(4):714–39. 10.1016/j.cmet.2018.03.004 29617640

[5] Tsimikas S. RNA-targeted therapeutics for lipid disorders. Curr Opin Lipidol (2018) 29(6):459–66. 10.1097/MOL.0000000000000549 30234555

[6] Nakamori M Junn E Mochizuki H Mouradian MM. Nucleic Acid-Based Therapeutics for Parkinson’s Disease. Neurotherapeutics (2019) 16(2):287–98. 10.1007/s13311-019-00714-7 PMC6554378 30756362

[7] Weng Y Xiao H Zhang J Liang XJ Huang Y. RNAi therapeutic and its innovative biotechnological evolution. Biotechnol Adv (2019) 37(5):801–25. 10.1016/j.biotechadv.2019.04.012 31034960

[8] Singh A Trivedi P Jain NK. Advances in siRNA delivery in cancer therapy. Artif Cells Nanomed Biotechnol (2018) 46(2):274–83. 10.1080/21691401.2017.1307210 28423924

[9] Laina A Gatsiou A Georgiopoulos G Stamatelopoulos K Stellos K. RNA Therapeutics in Cardiovascular Precision Medicine. Front Physiol (2018) 9:953. 10.3389/fphys.2018.00953 30090066 PMC6068259

[10] Goodman TT Olive PL Pun SH. Increased nanoparticle penetration in collagenase-treated multicellular spheroids. Int J Nanomed (2007) 2(2):265–74. PMC2673974 17722554

[11] Lee WH Loo CY Young PM Traini D Mason RS Rohanizadeh R. Recent advances in curcumin nanoformulation for cancer therapy. Expert Opin Drug Deliv (2014) 11(8):1183–201. 10.1517/17425247.2014.916686 24857605

[12] Tong X Wang Z Sun X Song J Jacobson O Niu G. Size Dependent Kinetics of Gold Nanorods in EPR Mediated Tumor Delivery. Theranostics (2016) 6(12):2039–51. 10.7150/thno.17098 PMC5039679 27698939

[13] Li C Penet MF Wildes F Takagi T Chen Z Winnard PT. Nanoplex delivery of siRNA and prodrug enzyme for multimodality image-guided molecular pathway targeted cancer therapy. ACS Nano (2010) 4(11):6707–16. 10.1021/nn102187v PMC2991391 20958072

[14] Chen Z Krishnamachary B Bhujwalla ZM. Degradable Dextran Nanopolymer as a Carrier for Choline Kinase (ChoK) siRNA Cancer Therapy. Nanomater (Basel) (2016) 6(2):34–41. 10.3390/nano6020034 PMC5302479 28344291

[15] Mehvar R. Dextrans for targeted and sustained delivery of therapeutic and imaging agents. J Controlled Release (2000) 69(1):1–25. 10.1016/S0168-3659(00)00302-3 11018543

[16] Gillies ER Goodwin AP Frechet JMJ. Acetals as pH-sensitive linkages for drug delivery. Bioconjugate Chem (2004) 15(6):1254–63. 10.1021/bc049853x 15546191

[17] Knorr V Russ V Allmendinger L Ogris M Wagner E. Acetal linked oligoethylenimines for use as pH-sensitive gene carriers. Bioconjugate Chem (2008) 19(8):1625–34. 10.1021/bc8001858 18627197

[18] Zhang Y Zhang GL Sun X Cao KX Ma C Nan N. Establishment of a murine breast tumor model by subcutaneous or orthotopic implantation. Oncol Lett (2018) 15(5):6233–40. 10.3892/ol.2018.8113 PMC5876452 29616105

[19] Ho KS Poon PC Owen SC Shoichet MS. Blood vessel hyperpermeability and pathophysiology in human tumour xenograft models of breast cancer: a comparison of ectopic and orthotopic tumours. BMC Cancer (2012) 12:579. 10.1186/1471-2407-12-579 23217114 PMC3539979

[20] Chen Z Krishnamachary B Penet MF Bhujwalla ZM. Acid-degradable Dextran as an Image Guided siRNA Carrier for COX-2 Downregulation. Theranostics (2018) 8(1):1–12. 10.7150/thno.21052 29290789 PMC5743456

[21] Longmire M Choyke PL Kobayashi H. Clearance properties of nano-sized particles and molecules as imaging agents: considerations and caveats. Nanomed (Lond Engl) (2008) 3(5):703–17. 10.2217/17435889.3.5.703 PMC3407669 18817471

[22] Seidel JA Otsuka A Kabashima K. Anti-PD-1 and Anti-CTLA-4 Therapies in Cancer: Mechanisms of Action, Efficacy, and Limitations. Front Oncol (2018) 8:86. 10.3389/fonc.2018.00086 29644214 PMC5883082

[23] Rao M Valentini D Dodoo E Zumla A Maeurer M. Anti-PD-1/PD-L1 therapy for infectious diseases: learning from the cancer paradigm. Int J Infect Dis (2017) 56:221–8. 10.1016/j.ijid.2017.01.028 28163164

[24] Sasikumar PG Ramachandra M. Small-Molecule Immune Checkpoint Inhibitors Targeting PD-1/PD-L1 and Other Emerging Checkpoint Pathways. BioDrugs (2018) 32(5):481–97. 10.1007/s40259-018-0303-4 30168070

[25] Guzik K Tomala M Muszak D Konieczny M Hec A Błaszkiewicz U. Development of the Inhibitors that Target the PD-1/PD-L1 Interaction-A Brief Look at Progress on Small Molecules, Peptides and Macrocycles. Molecules (2019) 24(11):2071–100. 10.3390/molecules24112071 PMC6600339 31151293

[26] Uhlén M Fagerberg L Hallström BM Lindskog C Oksvold P Mardinoglu A. Tissue-based map of the human proteome. Science (2015) 347(6220):1260419. 10.1126/science.1260419 25613900

[27] Michot JM Bigenwald C Champiat S Collins M Carbonnel F Postel-Vinay S. Immune-related adverse events with immune checkpoint blockade: a comprehensive review. Eur J Cancer (2016) 54:139–48. 10.1016/j.ejca.2015.11.016 26765102

[28] Gerson JN Ramamurthy C Borghaei H. Managing adverse effects of immunotherapy. Clin Adv Hematol Oncol (2018) 16(5):364–74. 29851932

[29] Feng J Yang H Zhang Y Wei H Zhu Z Zhu B. Tumor cell-derived lactate induces TAZ-dependent upregulation of PD-L1 through GPR81 in human lung cancer cells. Oncogene (2017) 36(42):5829–39. 10.1038/onc.2017.188 28604752

[30] Brin E Wu K Lu HT He Y Dai Z He W. PEGylated arginine deiminase can modulate tumor immune microenvironment by affecting immune checkpoint expression, decreasing regulatory T cell accumulation and inducing tumor T cell infiltration. Oncotarget (2017) 8(35):58948–63. 10.18632/oncotarget.19564 PMC5601705 28938609

[31] Xia X Zhou W Guo C Fu Z Zhu L Li P. Glutaminolysis Mediated by MALT1 Protease Activity Facilitates PD-L1 Expression on ABC-DLBCL Cells and Contributes to Their Immune Evasion. Front Oncol (2018) 8:632. 10.3389/fonc.2018.00632 30619766 PMC6305595

[32] Husain Z Huang Y Seth P Sukhatme VP. Tumor-Derived Lactate Modifies Antitumor Immune Response: Effect on Myeloid-Derived Suppressor Cells and NK Cells. J Immunol (2013) 191(3):1486–95. 10.4049/jimmunol.1202702 23817426

[33] Cascone T McKenzie JA Mbofung RM Punt S Wang Z Xu C. Increased Tumor Glycolysis Characterizes Immune Resistance to Adoptive T Cell Therapy. Cell Metab (2018) 27(5):977–87.e974. 10.1016/j.cmet.2018.02.024 29628419 PMC5932208

[34] Hu YM Hsiung YC Pai MH Yeh SL. Glutamine Administration in Early or Late Septic Phase Downregulates Lymphocyte PD-1/PD-L1 Expression and the Inflammatory Response in Mice With Polymicrobial Sepsis. JPEN J Parenter Enteral Nutr (2018) 42(3):538–49. 10.1177/0148607117695245 28633555

[35] Li X Wenes M Romero P Huang SC Fendt SM Ho PC. Navigating metabolic pathways to enhance antitumour immunity and immunotherapy. Nat Rev Clin Oncol (2019) 16(7):425–41. 10.1038/s41571-019-0203-7 30914826

[36] Gupta S Roy A Dwarakanath BS. Metabolic Cooperation and Competition in the Tumor Microenvironment: Implications for Therapy. Front Oncol (2017) 7:68. 10.3389/fonc.2017.00068 28447025 PMC5388702

[37] Clark CA Gupta HB Sareddy G Pandeswara S Lao S Yuan B. Tumor-Intrinsic PD-L1 Signals Regulate Cell Growth, Pathogenesis, and Autophagy in Ovarian Cancer and Melanoma. Cancer Res (2016) 76(23):6964–74. 10.1158/0008-5472.CAN-16-0258 PMC5228566 27671674

[38] Chang CH Qiu J O’Sullivan D Buck MD Noguchi T Curtis JD. Metabolic Competition in the Tumor Microenvironment Is a Driver of Cancer Progression. Cell (2015) 162(6):1229–41. 10.1016/j.cell.2015.08.016 PMC4864363 26321679

[39] Escors D Gato-Canas M Zuazo M Arasanz H Garcia-Granda MJ Vera R. The intracellular signalosome of PD-L1 in cancer cells. Signal Transduct Target Ther (2018) 3:26. 10.1038/s41392-018-0022-9 30275987 PMC6160488

[40] Dong P Xiong Y Yue J Hanley SJB Watari H. Tumor-Intrinsic PD-L1 Signaling in Cancer Initiation, Development and Treatment: Beyond Immune Evasion. Front Oncol (2018) 8:386. 10.3389/fonc.2018.00386 30283733 PMC6156376

[41] Kleffel S Posch C Barthel SR Mueller H Schlapbach C Guenova E. Melanoma Cell-Intrinsic PD-1 Receptor Functions Promote Tumor Growth. Cell (2015) 162(6):1242–56. 10.1016/j.cell.2015.08.052 PMC4700833 26359984

[42] Kwak G Kim D Nam GH Wang SY Kim IS Kim SH. Programmed Cell Death Protein Ligand-1 Silencing with Polyethylenimine-Dermatan Sulfate Complex for Dual Inhibition of Melanoma Growth. ACS Nano (2017) 11(10):10135–46. 10.1021/acsnano.7b04717 PMC5697980 28985469

[43] Kwak SY Lee S Han HD Chang S Kim KP Ahn HJ. PLGA Nanoparticles Codelivering siRNAs against Programmed Cell Death Protein-1 and Its Ligand Gene for Suppression of Colon Tumor Growth. Mol Pharm (2019) 16(12):4940–53. 10.1021/acs.molpharmaceut.9b00826 31651174

[44] Zhou Y-J Wan W-J Tong Y Chen M-T Wang D-D Wang Y. Stimuli-responsive nanoparticles for the codelivery of chemotherapeutic agents doxorubicin and siPD-L1 to enhance the antitumor effect. J Biomed Mat Res Part B: Appl Biomater (2019) 108:1710–24. 10.1002/jbm.b.34516 31746127

[45] Hassannia H Ghasemi Chaleshtari M Atyabi F Nosouhian M Masjedi A Hojjat-Farsangi M. Blockage of immune checkpoint molecules increases T-cell priming potential of dendritic cell vaccine. Immunology (2020) 159(1):75–87. 10.1111/imm.13126 31587253 PMC6904588

[46] Burri PH Hlushchuk R Djonov V. Intussusceptive angiogenesis: its emergence, its characteristics, and its significance. Dev Dyn (2004) 231(3):474–88. 10.1002/dvdy.20184 15376313

[47] Gerhardt H Golding M Fruttiger M Ruhrberg C Lundkvist A Abramsson A. VEGF guides angiogenic sprouting utilizing endothelial tip cell filopodia. J Cell Biol (2003) 161(6):1163–77. 10.1083/jcb.200302047 PMC2172999 12810700

[48] Kalluri R. Basement membranes: structure, assembly and role in tumour angiogenesis. Nat Rev Cancer (2003) 3(6):422–33. 10.1038/nrc1094 12778132

[49] Maeda H Nakamura H Fang J. The EPR effect for macromolecular drug delivery to solid tumors: Improvement of tumor uptake, lowering of systemic toxicity, and distinct tumor imaging in vivo. Adv Drug Deliv Rev (2013) 65(1):71–9. 10.1016/j.addr.2012.10.002 23088862

